# Primary subglottic BCOR family sarcoma masquerading as refractory asthma: a case report

**DOI:** 10.1097/RC9.0000000000000813

**Published:** 2026-08-07

**Authors:** Vipa Rath Marpukdee, Jitchai Kayankarnnavee, Nilnetre Mahathanaruk

**Affiliations:** a Department of Otolaryngology Head and Neck Surgery, Faculty of Medicine, Ramathibodi Hospital, Mahidol University, Bangkok, Thailand; b Department of Anatomical Pathology, Nakhon Pathom Hospital, Nakhon Pathom, Thailand

**Keywords:** airway obstruction, BCOR sarcoma, case report, refractory asthma, subglottic tumor

## Abstract

**Introduction and importance:**

BCOR family sarcomas are rare mesenchymal tumors, usually arising in bone or soft tissue, and involvement of the upper airway is exceedingly uncommon. Their nonspecific respiratory presentation can mimic asthma, leading to delayed diagnosis. This case highlights a primary subglottic BCOR sarcoma presenting as refractory asthma, emphasizing a rare site and a diagnostic pitfall.

**Case presentation:**

A 26-year-old woman presented with persistent wheezing and progressive dyspnea, which were initially treated as asthma with partial improvement. Imaging revealed a subglottic mass causing critical airway narrowing. The symptoms progressed to upper airway obstruction. An emergency tracheotomy was performed at the tertiary hospital prior to endoscopic resection. Histopathology and immunohistochemistry confirmed an undifferentiated BCOR family sarcoma. Treatment was started by consensus at a multidisciplinary team meeting, and neoadjuvant chemotherapy was given, but the tumor progressed. Endoscopic left hemilaryngectomy was attempted; positive margins necessitated a total laryngectomy. At the 3-year follow-up, the patient remained disease-free.

**Clinical discussion:**

Subglottic sarcomas are rare and often misdiagnosed due to overlapping asthma-like symptoms. Red flag signs, such as stridor, hoarseness, and poor response to bronchodilators, should prompt urgent imaging and endoscopic evaluation. Complete surgical resection is the cornerstone of treatment.

**Conclusion:**

This case emphasizes the need to consider upper airway tumors in patients with refractory asthma and the importance of increased awareness of the unusual presentation of a BCOR-family sarcoma of the upper airway. Early imaging and specialist referral can prevent diagnostic delay and reduce the need for extensive surgery.

## Introduction

BCOR family sarcomas are rare mesenchymal malignancies driven by BCOR gene alterations, typically arising in the bone or soft tissue of young individuals. Primary upper airway involvement is exceedingly uncommon, with only a few reported cases[1]. Airway tumors often present with nonspecific respiratory symptoms and may be misdiagnosed as asthma, delaying definitive treatment. This case describes a primary subglottic BCOR family sarcoma presenting as refractory asthma, highlighting a rare anatomical location and an important diagnostic pitfall. Recognition of atypical features, such as stridor and voice change, is critical for early identification of upper airway pathology. This case report is presented in line with the SCARE checklist[2].

HIGHLIGHTS

- Primary subglottic BCOR sarcoma is an exceptionally rare airway tumor.
- Airway tumors may mimic refractory asthma, delaying the diagnosis.
- Stridor and hoarseness are key signs of upper airway obstruction.
- Diagnosis requires imaging, endoscopy, and immunohistochemistry.
- Early diagnosis may allow the patient to undergo organ-preserving surgery, and a total laryngectomy will be necessary if the extent of the tumor requires it.

## Case presentation

A 26-year-old woman presented with progressive dyspnea over 5 months. She had a history of chronic respiratory symptoms that had been treated as allergic rhinitis, chronic rhinosinusitis, pneumonia, and asthma for over a year, with only partial or temporary relief. She had no significant allergies, no family history of malignancy, and no known genetic disorders. Prior medical treatments included bronchodilators, antibiotics, antihistamines, decongestants, steroids, and supportive therapies, without sustained improvement.

On physical examination, the patient exhibited severe hoarseness and intermittent stridor. Vital signs were stable. These findings suggested upper airway obstruction rather than lower airway disease.

**Figure 1.**
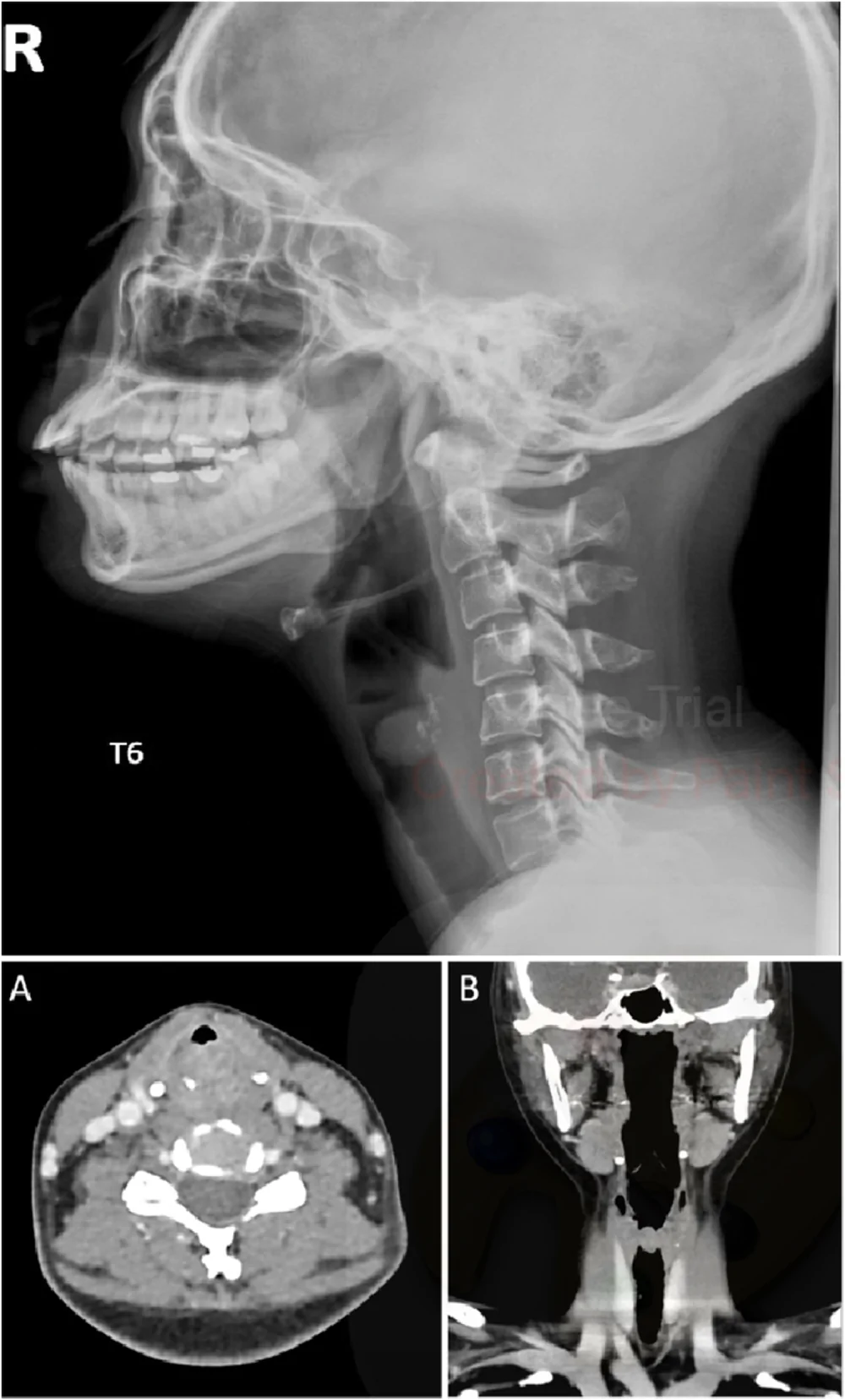
Top: Plain radiograph of the lateral neck showing a radiopaque mass attached to the posterior subglottic wall. Below: Computed tomography images showing a lobulated, infiltrative, enhancing mass at the posterior wall of the subglottis, causing severe narrowing of the laryngeal airway (A: axial view; B: coronal view).

**Figure 2.**
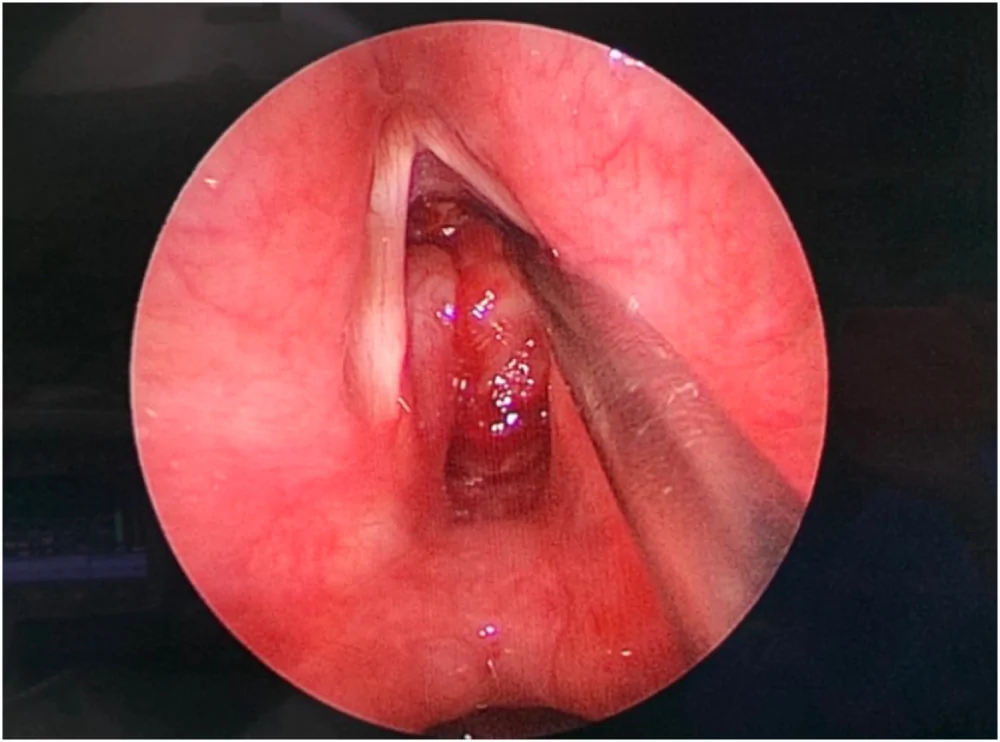
Endoscopic view of a lobulated submucosal subglottic mass causing almost complete airway occlusion.

**Figure 3.**
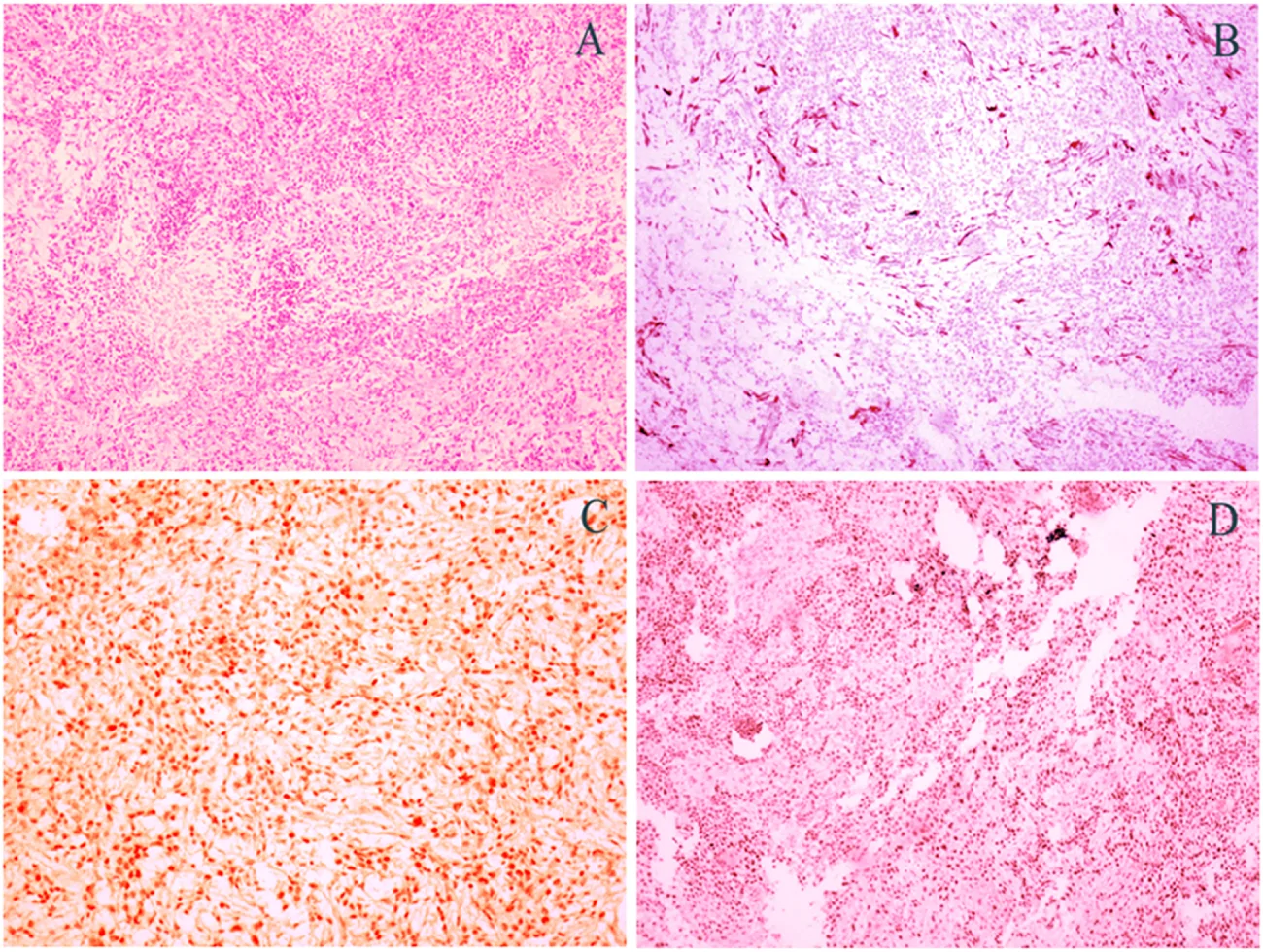
Tumor histopathology shows (A) a round-to-spindle-cell neoplasm suggestive of a mesenchymal tumor (hematoxylin and eosin; 100×). Immunohistochemistry was negative for (B) cytokeratins AE1 and AE3 and positive for (C) BCOR and (D) TLE1.

A lateral neck radiograph demonstrated a subglottic soft-tissue opacity. The patient was referred to a provincial hospital, where a CT revealed an enhancing submucosal mass causing severe airway narrowing (Fig. 1). She was then transferred to a tertiary hospital with an ENT service for further management. Her condition worsened, with stridor; an emergency awake tracheostomy was then performed at the level of the 3rd–4th tracheal rings to avoid tumor violation, and direct laryngoscopy confirmed a near-obstructing submucosal lesion (Fig. 2). Histopathological examination demonstrated a round-to-spindle-cell neoplasm suggestive of a mesenchymal tumor with positive margins. Immunohistochemistry showed positivity for TLE1, Bcl6, SATB2, BCOR, Factor XIIIa, SMA (focal), and INI-1, and negativity for epithelial, neural, and vascular markers (Fig. 3), most consistent with a rare BCOR-family tumor.

Multidisciplinary evaluation concluded with a diagnosis of a rare undifferentiated spindle/round cell BCOR-family sarcoma. Metastatic workup revealed no regional or distant disease; overall staging was T2N0M0, G2, based on the AJCC 8th edition. Given the patient’s young age, organ-preserving treatment was initially pursued. Due to a lack of standardized protocols, neoadjuvant VAC-IE chemotherapy (vincristine, doxorubicin, cyclophosphamide alternating with ifosfamide and etoposide), which was derived from other sarcomas, was administered; however, follow-up imaging demonstrated tumor progression. Endoscopic left hemilaryngectomy was attempted and involved removal of the true vocal cord, false vocal cord, paraglottic space, arytenoid, and subglottic mucosa on the affected side with at least a 2 mm margin, but positive margins, especially in the posterolateral region, necessitated conversion to total laryngectomy, achieving complete resection. The patient completed treatment, including adjuvant chemotherapy, without complications. No major adverse or unexpected events were reported. At 3-year follow-up, surveillance imaging and endoscopy showed no evidence of recurrence, good compliance with esophageal voice rehabilitation, no swallowing problems, and a normal social life.

## Discussion

Subglottic tumors are rare and exhibit anatomical and pathological variability. The differential diagnosis includes conditions ranging from benign to malignant, such as amyloidosis, sarcoidosis, tuberculosis infection, squamous cell carcinoma, lymphoma, and sarcomas^[^3–5^]^. Sarcomas in this region account for less than 1% of laryngeal malignancies[6]. Their presentation often mimics common airway diseases, such as asthma, leading to delayed diagnosis. Patients typically present with progressive dyspnea unresponsive to standard therapy, and diagnosis is frequently established only after imaging or endoscopic evaluation reveals airway obstruction. The patient’s prolonged history of asthma-like symptoms contributed to delayed diagnosis. Overlapping clinical features between asthma and central airway obstruction posed a diagnostic challenge.

Management of laryngeal sarcoma varies due to its rarity and lack of standardized guidelines. Surgical resection remains the mainstay of treatment, with approaches ranging from endoscopic excision to total laryngectomy, depending on tumor extent and response to therapy^[^3,4,6^]^. There is no evidence supporting organ-preserving surgery for BCOR sarcoma in the upper airway region, but such strategies are desirable, especially in young patients. Complete oncologic resection with negative margins is critical for optimal outcomes. In this case, initial chemotherapy failed to control tumor progression, necessitating definitive surgical management.

The role of radiotherapy in sarcoma is limited due to relative radioresistance, and it is typically reserved for selected high-risk cases. Our patient did not receive radiation because radiotherapy does not have an adjuvant role[7]. Chemotherapy responsiveness varies by histological subtype; BCOR family sarcomas are rare and may demonstrate unpredictable behavior. Samer *et al*[8] reported a complete pathologic response to chemotherapy (VAC-IE) in a BCOR sarcoma. In contrast, Merjaneh *et al*[9] showed that tumor response to chemotherapy varies, as seen in this case. Prognosis depends on factors such as age, tumor grade, histology, and completeness of surgical resection. At the 3-year follow-up, the patient has an excellent prognosis, and she acknowledges that being disease-free is important^[^10,11^]^. Evidence suggests that surgical treatment is associated with better outcomes than non-surgical approaches.

In this case, we emphasize the early recognition, diagnosis, and management of rare tumors such as BCOR sarcoma arising in rare anatomical regions such as the subglottic area. The role of neoadjuvant chemotherapy and complete surgical resection remains the cornerstone of treatment for localized disease. Postoperative outcomes, including disease-free survival and patient quality of life, are excellent.

## Conclusion

Subglottic sarcoma is an exceptionally rare entity that can mimic common respiratory conditions, leading to delayed diagnosis and potentially life-threatening airway obstruction. Early recognition through careful clinical assessment and appropriate imaging is essential. Management requires a multidisciplinary approach, with surgical resection playing a central role in achieving disease control. Increased awareness of this condition may facilitate earlier diagnosis and reduce the need for extensive surgical intervention. International registries are needed to define optimal chemotherapy regimens and the role of organ-preserving surgery for primary subglottic BCOR sarcoma.

## Ethical approval

The study was approved by the Ethics Committees of the Institutional Review Boards in Mahidol University under certificate of approval number COA. No. MURA2023/606.

## Consent

Written informed consent was obtained from the patient for publication of clinical details and images. All procedures were conducted in accordance with the Declaration of Helsinki.

## Sources of funding

None.

## Data Availability

The data supporting this case report are included within the article. Additional de-identified clinical information and imaging data are available from the corresponding author upon reasonable request, subject to ethical and patient confidentiality considerations
